# Urinary liver‐type fatty acid‐binding protein: A hemodynamic index during extracorporeal membrane oxygenation

**DOI:** 10.1002/ccr3.6531

**Published:** 2022-11-06

**Authors:** Ryo Ichibayashi, Saki Yamamoto, Yoshimi Nakamichi, Yuka Masuyama, Hibiki Serizawa, Masayuki Watanabe, Takayuki Yabe, Mitsuru Honda

**Affiliations:** ^1^ Department of Critical Care Center Toho University Medical Center Omori Hospital Ota‐ku Japan

**Keywords:** circulation index, extracorporeal membrane oxygenation, lactate, liver‐type fatty acid‐binding protein

## Abstract

We discuss a case in which urinary L‐FABP measurements were used to manage a 46‐year‐old male patient receiving V‐A ECMO support. His mean blood pressure was ≥75 mmHg for the first 24 h after the initiation of V‐A ECMO, and he experienced a rapid decrease in urinary L‐FABP levels.

## Graphical abstract

Measurement of urinary L‐FABP levels can aid in indicators of circulation and management of V‐A ECMO. Urinary L‐FABP levels may reflect a slight shortage of intravascular volume. And this study is suggested that urinary L‐FABP may be more sensitive than lactate in controlling intravascular water balance.

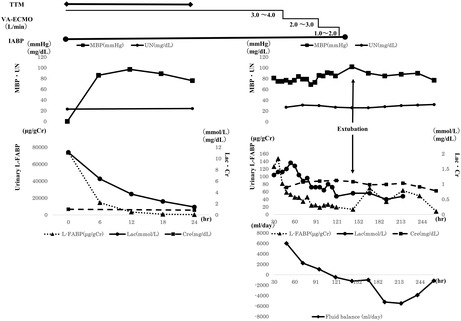

## 1 INTRODUCTION

Veno‐arterial extracorporeal membrane oxygenation (V‐A ECMO) is applied in circulatory failure cases such as cardiogenic shock, cardiac arrest, and refractory ventricular fibrillation. V‐A ECMO management includes echocardiographic assessments of cardiac function and evaluations of the peripheral circulation based on venous oxygen saturation, lactate levels, regional cerebral oxygen saturation, and central venous pressure. ECMO flow rates exceeding cardiac output result in low pulse pressure, leading to low mean blood pressure. Thus, the mean blood pressure is also used to manage organ perfusion and peripheral circulation during ECMO support.

Generally, urinary liver‐type fatty acid‐binding protein (L‐FABP) is a useful clinical marker in the monitoring of chronic kidney disease (CKD).^1^ And it can serve clinically as a predictive marker for contrast medium‐induced nephropathy and acute kidney injury.^2^, ^3^ In addition, urinary L‐FABP—a biomarker of renal ischemia—has also been considered to reflect acute systemic ischemic injury and to be effective in the assessment of sepsis treatment.^4^, ^5^ However, no reports have discussed urinary L‐FABP use during V‐A ECMO management in severe ischemic injury after cardiac arrest cases.

Herein, we discuss a case in which urinary L‐FABP measurements were used during V‐A ECMO management.

## 2 CASE HISTORY

Here, we describe a 46‐year‐old male patient who had collapsed at home in the presence of a close relative, who called for an ambulance and started resuscitation maneuvers until the arrival of the medical team. He has no medical history. The monitor waveform showed ventricular fibrillation requiring three defibrillation shocks, endo‐tracheal intubation, and transport to our hospital. Extracorporeal cardiopulmonary resuscitation was initiated on arrival due to the onset of refractory ventricular fibrillation. The interval between the onset of cardiac arrest and V‐A ECMO cannulation was 45 min. After starting V‐A ECMO, defibrillation was performed once. The monitor waveform became pulseless electrical activity (PEA).

Electrocardiographic changes and increased levels of creatine kinase‐muscle/brain (CK‐MB) were suggestive for myocardial infarction (Table 1). An emergency coronary angiogram revealed a significant stenosis of the left anterior descending coronary artery confirming the diagnosis of acute anterior wall myocardial infarction. The lesion was treated with percutaneous coronary intervention (PCI) followed by the insertion of an intra‐aortic balloon pump (IABP). After performing PCI, return of spontaneous circulation (ROSC) was confirmed. The patient was transferred to the intensive care unit (ICU) and treated with targeted temperature management (TTM) at 34°C for 24 h. Subsequently, the patient's body temperature was gradually restored to 36°C over the next 48 h.

**TABLE 1:** Laboratory results on admission

*Biochemical*			*Coagulation*		
TP	6.4	g/dl	APTT	35.3	s
Alb	3.6	g/dl	PT	14.1	s
CK	139	U/L	PT‐INR	1.1	
CK‐MB	77	U/L	PT%	81	%
AST	119	U/L	FDP	31	μg/ml
ALT	70	U/L			
LD	450	U/L	*Venous blood gas*		
ALP	238	U/L	pH	7.034	
γ‐GT	70	U/L	PCO_2_	84.5	Torr
Cre	1.01	mg/dl	PO_2_	15.1	Torr
UN	23	mg/dl	Lac	11.1	mmol/L
eGFR	64	ml/min/1.73 m^2^			
NH_3_	179	μg/dl	*Immunological*		
Glu	307	mg/dl	CRP	0.1	mg/dl
HbA1c	5.1	%	TnI	0.03	or less pg/ml
Na	136	mEq/L			
K	4.7	mEq/L	*Urinalysis*		
Cl	100	mEq/L	L‐FABP	74,100	μg/gCr
*Hematology*					
WBC	9800	/μl			
Hb	17.5	g/dl			
Plt	26.1	×10^4^/μl			

Abbreviations: Alb: albumin; ALP: alkaline phosphatase; ALT: alanine aminotransferase; APTT: activated partial thromboplastin time; AST: aspartate aminotransferase; Cre: creatinine; CK: creatine kinase; CRP: C‐reactive protein; eGFR: estimated glomerular filtration rate; FDP: fibrinogen degradation product; Glu: glucose; Hb; hemoglobin; INR: international normalized ratio; L‐FABP: liver‐type fatty acid‐binding protein; Lac: lactate; LD: lactate dehydrogenase; PT: prothrombin time; Plt: platelet; TP: total protein; TnI: troponin I; UN: urea nitrogen; WBC: white blood cell; γ‐GT: gamma‐glutamyltransferase.

**FIGURE 1:**
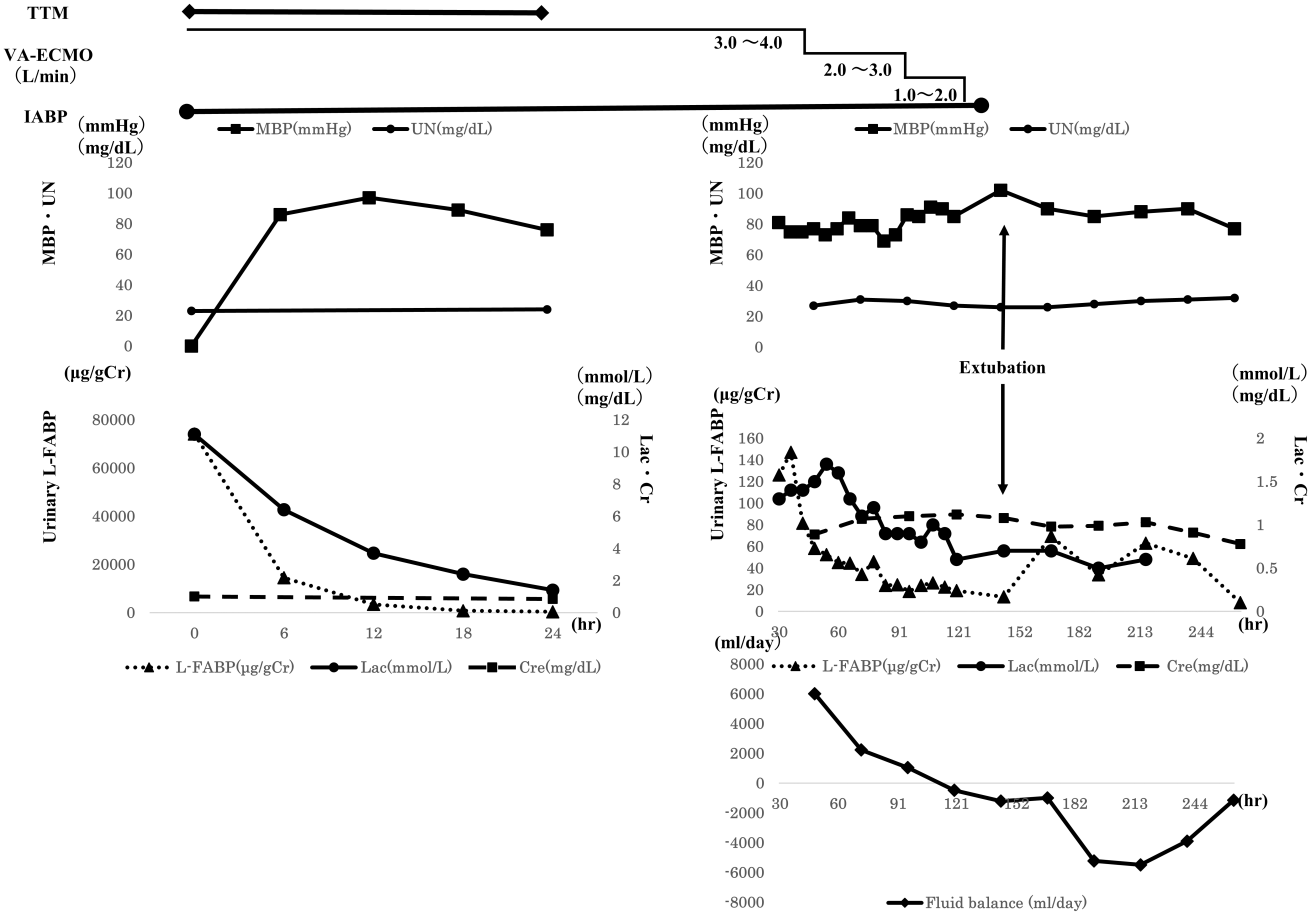
Clinical course. Lactate and urinary L‐FABP levels decreased with stabilization of hemodynamics. While the daily fluid balance was negative, urinary L‐FABP levels remained mildly elevated. TTM: targeted temperature management; MBP: mean blood pressure; UN: urea nitrogen; V‐A ECMO: veno‐arterial extracorporeal membrane oxygenation; L‐FABP: liver‐type fatty acid‐binding protein; IABP: intra‐aortic balloon pumping; Lac: lactate; Cre: creatinine.

Figure 1 shows changes in urinary L‐FABP, lactate, UN, Cre, mean blood pressure, daily fluid balance, flow rate, and other parameters during ICU admission. Values measured from urine collected before ECMO cannulation on arrival were regarded as baseline values, and urinary L‐FABP and serum lactate levels were measured every 6 h. Urinary L‐FABP levels were also measured every 24 h after weaning. On arrival, the patient's urinary L‐FABP level was 74,100 μg/gCr, which decreased to 816 μg/gCr at 24 h after admission, at which time the lactate level, which had also been high, decreased to 2.4 mmol/L. Twenty‐four hours after arrival, the ECMO flow rate was 2.6–3.6 L/min; mean blood pressure, 76–97 mmHg; and pulse pressure, 42–60 mmHg. On and after hospital day 2, the patient's lactate levels remained at 0.9–1.0 mmol/L. However, his mean blood pressure increased, while urinary L‐FABP levels decreased.

On hospital day 5, the patient was weaned off V‐A ECMO without deterioration of urinary L‐FABP levels, lactate levels, or mean blood pressure. On hospital day 6, he was weaned off IABP without changes in mean blood pressure or lactate levels. Thereafter, although the mean blood pressure remained unchanged, urinary L‐FABP levels temporarily increased, following which they decreased again. During the same period, the daily fluid balance was negative on consecutive days. During treatment in the ICU, UN and Cre values were within normal ranges. On hospital day 8, he could follow instructions and was weaned off mechanical ventilation and extubated on hospital day 10. On hospital day 13, he was discharged from the ICU and transferred to the general ward. On hospital day 24, no neurological sequelae were noted, and he was discharged from our hospital with independent gait.

## 3 DISCUSSION

Urinary L‐FABP is used to monitor CKD and predict the onset of acute kidney injury. Like the lactate value, it is also used to determine microcirculatory disturbances.^4^ Lactate levels increase owing to tissue hypoxia resulting from circulatory failure or insufficient oxygen supply. Therefore, improving circulation or oxygenation should decrease lactate levels. However, some patients with normal hemodynamics and oxygenation exhibit elevated lactate levels due to the influence of underlying diseases such as sepsis and malignant tumors.^6^ Such elevation may be attributed to increased lactate production, decreased lactate consumption, or both in various organs such as the muscles, skin, and brain. Contrastingly, FABPs are specifically distributed in various tissues.^7^ For example, urinary L‐FABP, which is released from proximal tubule cells, has been highlighted as a strong biomarker of acute ischemic injury.^4^ Given that urinary L‐FABP is unlikely to be affected by other factors, it may represent a more sensitive hemodynamic indicator than lactate.

During ECMO, the mean blood pressure is often maintained at ≥65 mmHg. However, there are no clear criteria for the index value, and the target value varies across institutions. Although one report suggested a higher survival rate among patients managed with a target mean blood pressure of 70–80 mmHg,^8^ the optimal mean blood pressure is considered to differ for each patient. Urinary L‐FABP levels should decrease if patients are managed with optimal mean blood pressure, suggesting they can aid in blood pressure management.

Our patient's mean blood pressure was ≥75 mmHg for the first 24 h after V‐A ECMO initiation, and he experienced rapid decreases in both urinary L‐FABP and lactate levels. In this case, the target mean blood pressure was 70 mmHg, and weaning from V‐A ECMO was eventually successful.

In patients managed with a mean blood pressure of ≥70 mmHg who exhibit normal lactate levels, temporary increases in urinary L‐FABP occurred following extubation, as observed in our patient. One possible explanation is that the daily fluid balance was adjusted between −500 and −5000 ml during this period to improve systemic edema. Therefore, the volume of fluid replacement is maintained at the minimum level to ensure a relatively higher urinary output. In general, variation in the UN/Cre ratio is one way of measuring the degree of intravascular dehydration. However, there was no significant increase in the ratio during this period. Thus, urinary L‐FABP levels may reflect a slight shortage of intravascular volume that is not reflected by lactate levels and UN/Cre ratio. We believe that the measurement of L‐FABP is one of the useful tests that can determine the fluid balance of critically ill patients.

On the contrary, urinary L‐FABP measurement has a defect. Since urinary L‐FABP levels are measured from urine samples, they cannot be used for patients undergoing dialysis or those with anuria in the early stage of shock. And Lactate levels detected by blood gas tests can be measured within 1 min, whereas urinary L‐FABP measurements take 10 min. For this reason, urinary L‐FABP is not an emergency indicator that requires results within minutes.

We routinely measure urinary L‐FABP in critically ill patients treated with ECMO. Circulatory management is performed by comprehensively examining parameters such as lactic acid level and blood pressure, and urinary L‐FABP. Unfortunately, urinary L‐FABP failed to prove to be more sensitive than traditional hemodynamic indicators such as lactate and mean blood pressure. But urinary L‐FABP is measured from urine samples. As a strength of the current inspection, urinary L‐FABP is to be a non‐invasive hemodynamic indicator compared with lactate as determined by blood tests. It also may be useful as an index of hemodynamics in the progression of anemia or in patients without invasive arterial pressure measurements.

## 4 CONCLUSION

Our findings suggest that urinary L‐FABP can be used as a hemodynamic indicator for managing patients receiving V‐A ECMO support and it can be used equivalently to conventional hemodynamic indicators such as lactate and mean blood pressure. And this study is suggested that urinary L‐FABP may be more sensitive than lactate in controlling intravascular water balance. But, since it is a case study, it is necessary to accumulate and investigate cases in the future.

## AUTHOR CONTRIBUTIONS

RI wrote and drafted the manuscript. SY and YM measured urinary L‐FABP and lactate levels. YN, HS, MW, TY, and MH drafted the manuscript. All authors read and approved the final manuscript.

## FUNDING INFORMATION

This research did not receive any specific grant from funding agencies in the public, commercial, or not‐for‐profit sectors.

## ETHICS STATEMENT

The present study was approved by the Toho University Medical Center Omori Hospital Ethics Committee. Informed consent was obtained from participants or their family members prior to enrollment and after receiving written explanations about the aims and procedures used in the study (Approval No. M19141).

## CONSENT

Written informed consent was obtained from the patient for publication of this case report.

## Data Availability

The data that support the findings of this study are available from the corresponding author upon reasonable request.
